# Comparative mitogenomic analysis of Chinese cavefish *Triplophysa* (Cypriniformes: Nemacheilidae): novel gene tandem duplication and evolutionary implications

**DOI:** 10.1186/s12864-025-11486-0

**Published:** 2025-03-24

**Authors:** Shuang Song, Jianhan Cao, Hongmei Xiang, Zhixiao Liu, Wansheng Jiang

**Affiliations:** 1 College of Biology and Environmental Sciences, Jishou University, Jishou, 416000 China; 2 National and Local United Engineering Laboratory of Integrative Utilization Technology of Eucommia ulmoides, Jishou University, Zhangjiajie, 427000 China

**Keywords:** Cave fish, Mitogenome, Phylogeny, Positive selection, Karst, China

## Abstract

**Background:**

Cavefish exhibit significant morphological changes that result in trade-offs in metabolic requirements and energy utilization in perpetual darkness. As cellular “powerhouses”, mitochondria play crucial roles in energy metabolism, suggesting that mitochondrial genes have likely experienced selective pressures during cavefish evolution.

**Results:**

This study presents the first assembly of the complete mitogenome of *Triplophysa yangi*, a typical cavefish species in China. The mitogenome is 17,068 bp long, marking the longest recorded for the genus *Triplophysa*, and includes 13 protein-coding genes (PCGs), 2 rRNAs, 25 tRNAs, and a noncoding control region. An ~ 500 bp insertion between ND2 and WANCY regions was observed, comprising a large intact tandem repeat unit (A’-N’-OL’-C’) flanked by two unannotated sequences (U1/U2). The evolutionary origin of this repeat unit may involve either in situ duplication events with subsequent functional divergence—where neofunctionalization, subfunctionalization, or pseudogenization drove differential mutation rates between paralogs—or alternatively, horizontal acquisition from exogenous genetic material that became functionally integrated into the ancestral *T. yangi* mitogenome through co-option mechanisms. Phylogenetic analyses revealed two major clades within *Triplophysa*—epigean and hypogean lineages—consistent with previous classifications, while cave-restricted species exhibited signs of parallel evolution within the hypogean lineage. Selective pressure analysis indicated that the hypogean lineage (cave-dwelling groups, II & III) have a significantly increased ratio of nonsynonymous to synonymous substitution rates (ω) compared to the epigean lineage (surface-dwelling group, I), suggesting a combination of adaptive selection and relaxed functional constraints in cave-dwelling species.

**Conclusions:**

The duplication of tRNAs in *T. yangi* and the potential positive selection sites identified in *Triplophysa* cavefish further indicated adaptive evolution in mitochondrial PCGs in response to extreme subterranean conditions.

**Supplementary information:**

The online version contains supplementary material available at 10.1186/s12864-025-11486-0.

## Background

Cavefish, a specialized group that spends much or all of their lives in subterranean river habitats [1], has emerged as excellent models in developmental and evolutionary biology [2]. Over 230 cavefish species have been discovered worldwide, providing unique insights into how organisms adapt to extreme environments [3]. Notable morphological changes in cavefish include regressive traits, such as eye- and pigmentation loss, which arise convergently across different evolutionary lineages. Additionally, constructive sensory adaptations, such as increased neuromasts and taste buds, are frequently observed [4, 5]. Adaptive evolution in cavefish also involves metabolic changes, including enhanced fat-synthesis pathways [6] and the regulation of sugar metabolism and antioxidant mechanisms [7]. Overall, these changes represent trade-offs in metabolic requirements and energy utilization during cavefish adapt to perpetual darkness [8].

**Fig. 1:**
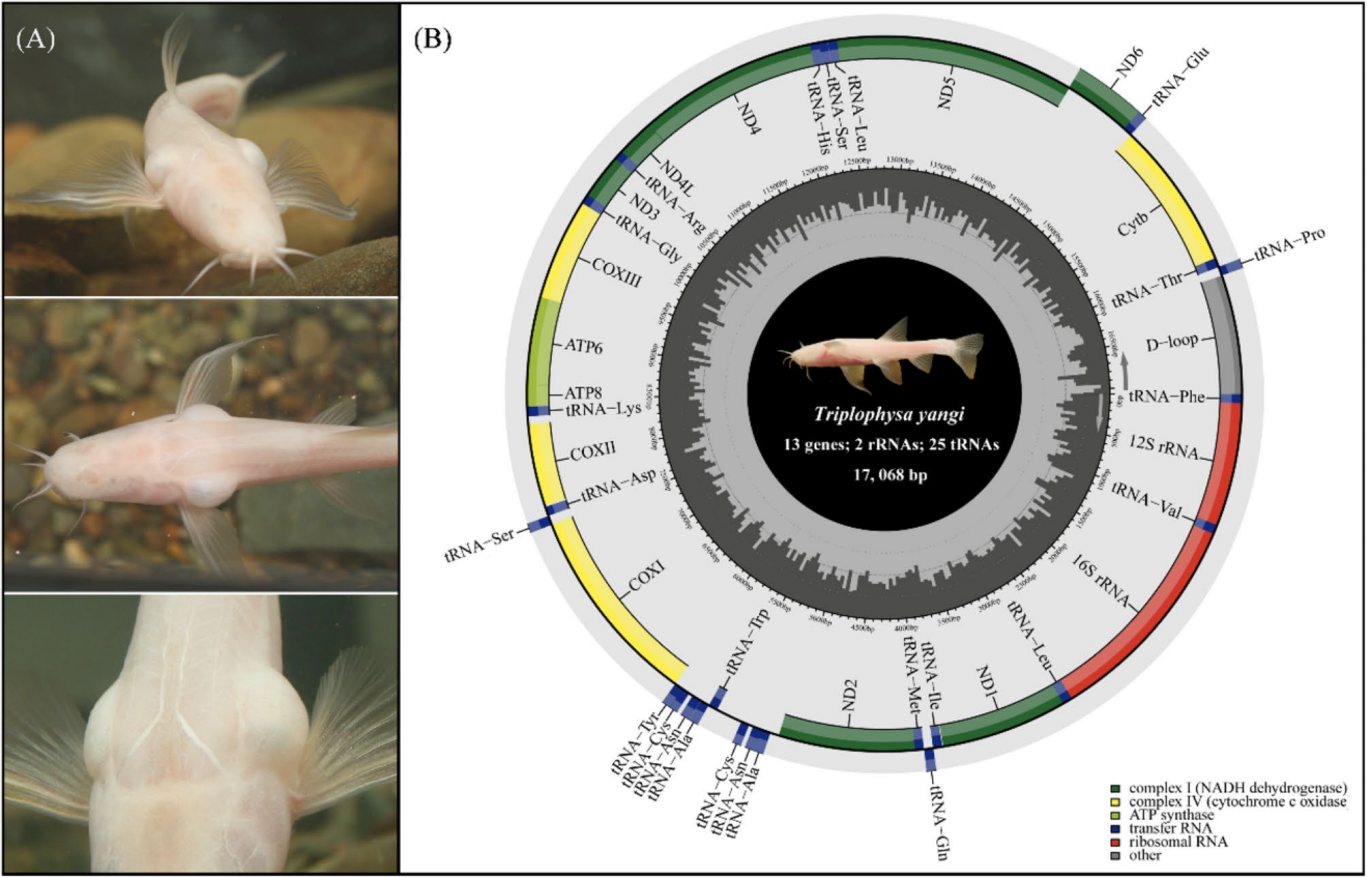
Live photos of *T. yangi* (**A**, showing the extraordinarily enlarged swim bladder chambers that protrude beyond the bilateral body wall resembling a fish with " flotation device”) and gene map of its newly sequenced mitogenome (**B**)

The karst landscape in Southwest China, covering approximately 2.4 million square kilometers, is home to over 150 cavefish species, representing the most diverse cavefish fauna on the earth [9]. Among the cavefish genera in China, the hypogean lineage of *Triplophysa* loaches ranks as the second largest group, surpassed only by *Sinocyclocheilus* cyprinids (approximately 40 vs. 70 species [10, 11]). Like *Sinocyclocheilus* cavefish [12], the hypogean lineage of *Triplophysa* exhibits remarkable morphological diversity, ranging from semi-cave-dwelling morphs with reduced eyes and pigmentation to fully cave-restricted morphs that are usually blind and white. Notably, some unusual morphological traits have also evolved in the hypogean lineage of *Triplophysa*. Recently, we described a new species, *Triplophysa yangi*, collected from a subterranean river in Shizong County, Yunnan, China [13]. This species features extraordinarily enlarged swim bladder chambers that protrude beyond the bilateral body wall, resembling a fish with a kind of “flotation device” (Fig. 1A). We proposed that this unique trait represents a novel troglomorphic adaptation in *Triplophysa* cavefish, in addition to the typical characteristics of eye reduction and lack of pigmentation.

Mitochondria, frequently termed cellular “powerhouses” or “energy factories”, drive critical metabolic processes—most notably ATP synthesis via oxidative phosphorylation (OXPHOS) to meet cellular energy demands. Although mitochondrial sequences have been widely used in phylogenetic studies owing to their simple structure, conserved coding regions, rapid evolutionary rates, and maternal inheritance patterns [14, 15], molecular adaptation mechanisms in mitochondrial OXPHOS genes remain understudied [8]. Notably, while nuclear DNA encodes ~ 80% of OXPHOS enzymes (compared to mitochondrial DNA’s 13 subunits), these dual-origin components exhibit coordinated evolution and functional integration to maintain respiratory efficiency [16]. Intriguingly, energy-intensive species like bats demonstrate elevated selection pressures on mitochondrial-encoded OXPHOS genes relative to their nuclear counterparts. Nevertheless, both mitochondrial and nuclear OXPHOS genes experience greater selective pressures than nuclear-coded nonrespiratory genes [17].

As a unique biological group thriving in harsh environments devoid of light and with limited food, cavefish exhibit a range of metabolic changes distinct from their surface counterparts [6, 7]. It is reasonable to speculate that cavefish mitochondrial OXPHOS genes have undergone specific changes under evolutionary pressures in dark environments to facilitate unique energy strategies through the respiratory chain. Our previous comparative analysis of the mitogenomes of *Sinocyclocheilus* indicated that cave-dwelling species accumulated more nonsynonymous mutations in their mitochondrial PCGs than surface-dwelling species [8]. Another study involving 44 *Triplophysa* cavefish species also showed greater selective pressures in cavefish compared to non-cavefish species [18]. Since the first mitogenome of the hypogean lineage of *Triplophysa* was sequenced in 2012 (*Triplophysa rosa* [19]), an increasing number of mitogenomes have been sequenced and reported in GenBank, providing an excellent opportunity to examine the differing selective pressures on mitochondrial PCGs within this unique group.

In this study, we first assembled and annotated the complete mitogenome of the typical cave-dwelling species, *T. yangi*, and conducted a detailed examination of its characteristics. We then integrated this mitogenome with all available *Triplophysa* mitogenomes and performed clustering analysis using Principal Component Analysis (PCA) and average nucleotide identity (ANI) based on data from 49 *Triplophysa* species. Additionally, we reconstructed a phylogenetic tree using the mitochondrial PCG dataset and identified potential signals of positive selection in the mitochondrial PCGs of cave-dwelling species compared to their surface-dwelling counterparts. This study aims to provide insights into the mitogenomic evolution of *Triplophysa* cavefish that thrive in subterranean environments.

## Results

### Structures and characteristics of the mitogenome of *T. yangi*

The complete mitogenome of *T. yangi* was 17,068 bp in length, composed of 13 typical vertebrate PCGs, 2 rRNAs, 25 tRNAs, and a noncoding control region (Fig. 1B). Most genes were encoded on the heavy (H) strand, with the exception of ND6 and eleven tRNA genes encoded on the light (L) strand. Among the PCGs, only ND1 and COI used GTG as their start codon, while all other start codons were ATG. The stop codons in the PCGs included: ND1, ND2, COI, ATP6, ATP8, ND4L, ND5, and ND6, which ended with TAA; ND3, which ended with TAG; and COII, COIII, and CYTB, which used an incomplete T (--), while ND4 employed another incomplete TA (-) (Table 1).

**Table 1:** Mitochondrial genome organization of *T. Yangi*

Gene	Position		Length(bp)	Codon		Intergenic nucleotide	Strand	Anticodon
	From	to		Start	Stop			
tRNA^Phe^	1	69	69			0	H	AAG
12 S rRNA	70	1018	949			2	H	
tRNA^Val^	1021	1092	72			20	H	CAU
16 S rRNA	1113	2768	1656			0	H	
tRNA^Leu^	2769	2843	75			6	H	AAU
ND1	2844	3818	975	GTG	TAA	0	H	
tRNA^Ile^	3825	3896	72			–2	H	UAG
tRNA^Gln^	3895	3965	71			1	L	GUU
tRNA^Met^	3967	4035	69			0	H	UAC
ND2	4036	5082	1047	ATG	TAA	66	H	
tRNA^Ala^	5149	5217	69			1	L	CGU
tRNA^Asn^	5219	5289	71			2	L	UUG
OL	5292	5322	31			–2	H	
tRNA^Cys^	5321	5385	65			206	L	ACG
tRNA^Trp^	5592	5661	70			2	H	ACU
tRNA^Ala^	5664	5731	68			1	L	CGU
tRNA^Asn^	5733	5805	73			2	L	UUG
OL	5808	5838	31			–2	H	
tRNA^Cys^	5837	5897	61			–1	L	ACG
tRNA^Tyr^	5897	5964	68			1	L	AUG
COI	5966	7516	1551	GTG	TAA	0	H	
tRNA^Ser^	7517	7587	71			1	L	AGU
tRNA^Asp^	7589	7660	72			12	H	CUG
COII	7673	8363	691	ATG	T(--)	–1	H	
tRNA^Lys^	8363	8438	76			1	H	UUU
ATPase8	8440	8607	168	ATG	TAA	–10	H	
ATPase6	8598	9281	684	ATG	TAA	–1	H	
COIII	9281	10,064	784	ATG	T(--)	0	H	
tRNA^Gly^	10,065	10,138	74			0	H	
ND3	10,139	10,489	351	ATG	TAG	–2	H	
tRNA^Arg^	10,488	10,557	70			0	H	GCU
ND4L	10,558	10,854	297	ATG	TAA	–7	H	
ND4	10,848	12,229	1382	ATG	TA(-)	0	H	
tRNA^His^	12,230	12,298	69			0	H	GUG
tRNA^Ser^	12,299	12,365	67			1	H	UCG
tRNA^Leu^	12,367	12,438	72			0	H	GAU
ND5	12,439	14,277	1839	ATG	TAA	–4	H	
ND6	14,274	14,795	522	ATG	TAA	0	L	
tRNA^Glu^	14,796	14,864	69			4	L	CUU
CYTB	14,869	16,009	1141	ATG	T(--)	0	H	
tRNA^Thr^	16,010	16,082	73			–2	H	UGU
tRNA^Pro^	16,081	16,150	70			5	L	GGU
D-Loop	16,156	16,898	743			170	H	

**Fig. 2:**
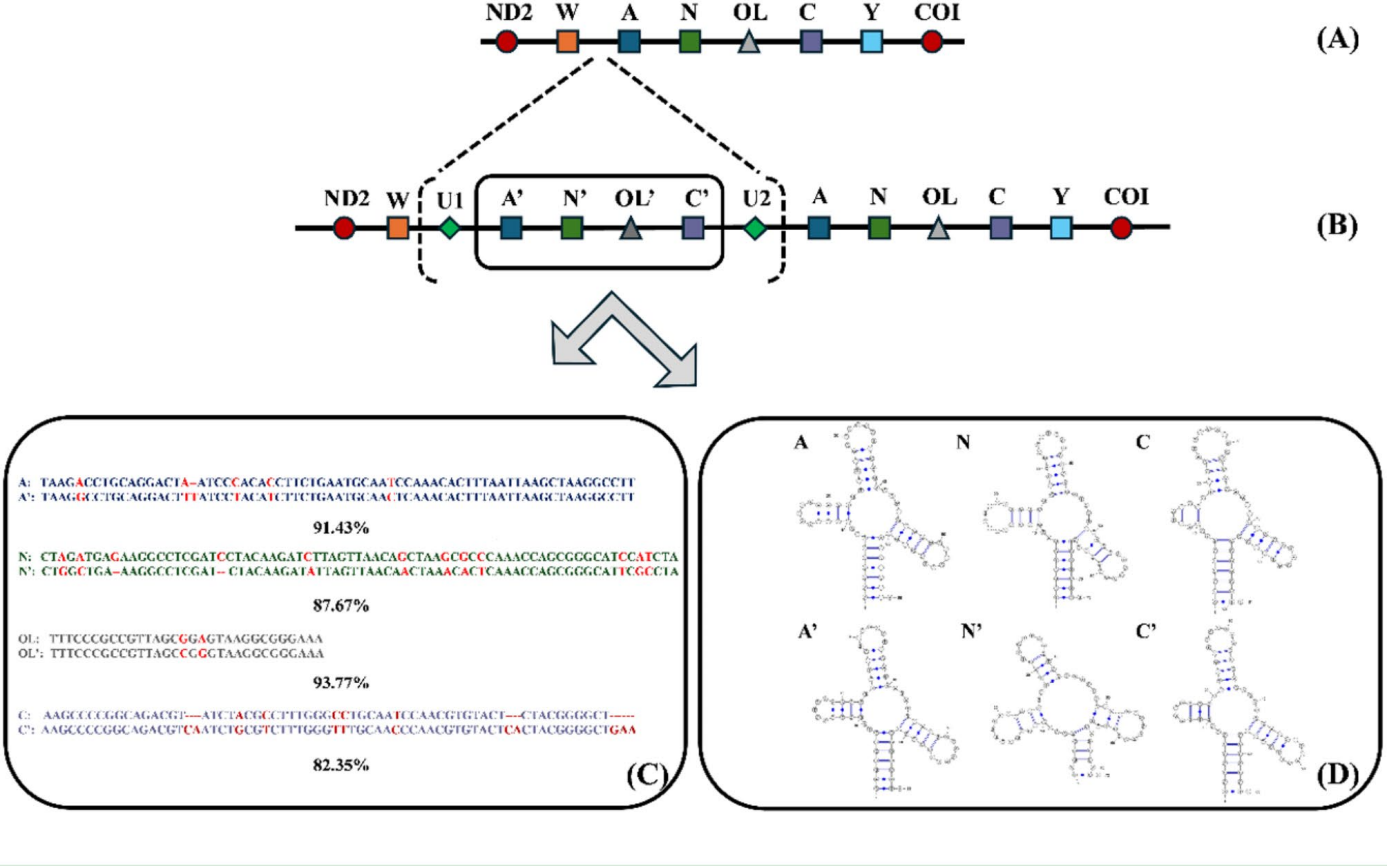
Gene rearrangement in the mitogenome of *T. yangi*. Notes: (**A**) The typical mitochondrial arrangement in *Triplophysa*; (**B**) The fragment insertion and gene duplication of *T. yangi*. (**C**) Sequence alignments and similarities between the original and duplicated copies of *T. yangi*. (**D**) Secondary structures of the original and duplicated copies of tRNAs. Abbreviations: A’: duplicated tRNA^Ala^; N’: duplicated tRNA^Asn^; C’: duplicated tRNA^Cys^; OL’: duplicated OL; U1: the unannotated forward flanking sequence; U2: the unannotated backward flanking sequence

Notably, the mitogenome of *T. yangi* contained 25 tRNA genes, distinguishing it from all other known species within the genus *Triplophysa*, which typically have 22 tRNAs. This increase was due to three duplicate copies of tRNA^Ala^ (A’), tRNA^Asn^ (N’), and tRNA^Cys^ (C’). Additionally, an OL (Origin of Light Strand Replication) copy (OL’) was also duplicated between tRNA^Asn^ (N’) and tRNA^Cys^ (C’), resulting in a large intact tandem repeat unit (A’-N’-OL’-C’, Fig. 2A) that maintained the same order as the original sequence (A-N-OL-C). The original unit (A-N-OL-C) was distinguished from the duplicated one (A’-N’-OL’-C’) based on parsimony and similarity: the original unit adhere to the conserved mitochondrial gene arrangement in *Triplophysa* species, and their sequences exhibit higher identity to orthologous genes in the closely related species. Interestingly, two unannotated sequences were located at the both ends of this A’-N’-OL’-C’ repeat unit. The unannotated forward flanking sequence (U1) was 66 bp in length and located between the stop codon of the original ND2 gene and the start of the A’-N’-OL’-C’ repeat unit. The unannotated backward flanking sequence (U2) was 206 bp, positioned between the end of the A’-N’-OL’-C’ repeat unit and the start of the original tRNA^Trp^. Both U1 and U2 did not match any full-coverage sequences when analyzed using BLAST against the NCBI “nucleotide collection (nr/nt)” database, even with the least rigorous algorithm. However, the last third of U2 (approximately 55 bp) matched the ND2 gene of other *Triplophysa* species with about 80% similarity.

Sequence similarities of the identified tRNAs and OL repeats were calculated after alignment with the original copies in the mitogenome of *T. yangi*. The duplicated tRNA^Ala^ (A’), tRNA^Asn^ (N’), OL’, and tRNA^Cys^ (C’) showed similarities of 91.43%, 87.67%, 93.77%, and 82.35% with their corresponding original copies (A, N, OL, and C) in *T. yangi*, respectively (Fig. 2B). Further analysis of the secondary structure of tRNAs indicated that the duplicated tRNAs (A’, N’, and C’) exhibited structural differences relative to the original copies (A, N, and C), particularly significant for tRNA^Asn^ (N’ vs. N, Fig. 2C).

### Sequence characteristics analysis of *Triplophysa* species

**Fig. 3:**
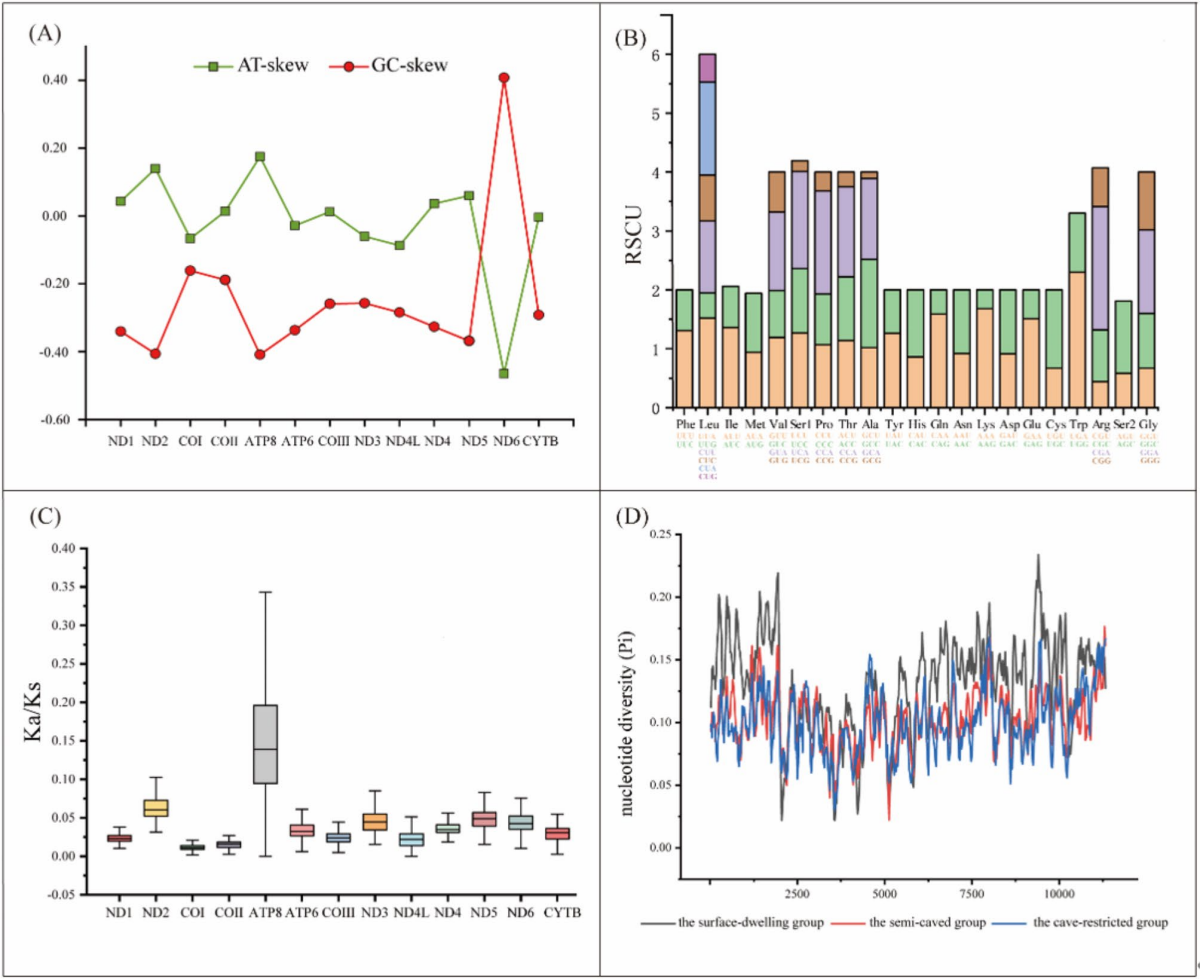
The characteristics of the mitogenome of *T. yangi* within the context of other congeners. Notes: Pictures show the GC and AT skews (**A**) and relative synonymous codon usage (RSCU) of *T. yangi* (**B**), the Ka/Ks ratio of 13 PCGs among 29 species of *Triplophysa* (**C**) and the nucleotide diversities according different groups (**D**)

The mitogenome base composition of *T. yangi* was as follows: T (27.5%), C (25.7%), A (30.4%), and G (16.4%), resulting in a high A + T content of 57.9%. The overall AT-skew was positive (0.05), while the GC-skew was negative (–0.22) (Table 2). Among the 13 PCGs, ND2, COII, ATP8, ND4, and ND5 exhibited positive AT-skews, whereas the others showed slight negative values, with ND6 displaying a marked decrease. All PCGs presented negative GC-skews, except for ND6, which showed a relatively high positive value (Fig. 3A). The RSCU values indicated that Leu was encoded by the greatest number of synonymous codons (*n* = 6), while Val, Ser1, Pro, Thr, Ala, Arg, and Gly were encoded by four codons each, and all remaining amino acids were encoded by only two codons (Fig. 3B).

**Table 2:** Base compositions (in percentages) of the mitogenomes that used for phylogenetic analysis

	**Species**	**Total length (bp)**	**T (%)**	**C (%)**	**A (%)**	**G (%)**	**A + T content (%)**	**AT-skew**	**GC-skew**	**Accession number**		
1	*T. aliensis*	16,565	28.5	25.3	27.8	18.5	56.3	–0.01	–0.16		KJ739868	
2	*T. alticeps*	16,572	28.8	25.1	28.2	17.9	57.0	–0.01	–0.17		MZ325251	
3	*T. angeli*	16,569	28.6	25.8	27.0	18.6	55.6	–0.03	–0.16		KT213584	
4	*T. anterodorsalis*	16,567	28.6	25.7	27.4	18.4	56.0	–0.02	–0.17		KT213585	
5	*T. baotianensis*	16,576	27.6	25.5	30.8	16.1	58.4	0.05	–0.23		MT992550	
6	*T. bleekeri*	16,568	28.6	25.8	27.1	18.5	55.7	–0.03	–0.16		JX135578	
7	*T. brevicauda*	16,572	28.2	25.6	27.9	18.3	56.1	–0.01	–0.17		KT213588	
8	*T. chondrostoma*	16,571	28.3	25.3	28.3	18.1	56.6	0.00	–0.17		KT213589	
9	*T. cuneicephala*	16,568	28.8	25.2	28.2	17.8	57.0	–0.01	–0.17		KY945352	
10	*T. dalaica*	16,576	28.2	25.6	28.3	17.9	56.5	0.00	–0.18		KT213590	
11	*T. dorsalis*	16,576	28.4	25.6	28.2	17.8	56.6	0.00	–0.18		KT213591	
12	*T. erythraea*	16,572	27.0	26.0	31.2	15.8	58.2	0.07	–0.24		PQ040451	
13	*T. fengshanensis*	16,607	27.0	25.9	31.3	15.8	58.3	0.07	–0.24		OQ998929	
14	*T. grahami*	16,566	28.5	25.0	29.2	17.5	57.7	0.01	–0.18		PP114297	
15	*T. hsutschouensis*	16,571	28.4	25.6	27.3	18.7	55.7	–0.02	–0.16		KT213592	
16	*T. huapingensis*	16,570	27.5	25.3	31.5	15.7	59.0	0.07	–0.23		OQ998930	
17	*T. jianchuanensis*	16,569	27.2	26.6	28.3	17.8	55.5	0.02	–0.20		OQ603602	
18	*T. labiata*	16,573	28.4	25.6	28.1	17.8	56.5	–0.01	–0.18		OQ559481	
19	*T. lixianensis*	16,570	28.5	25.4	27.8	18.4	56.3	–0.01	–0.16		KT966735	
20	*T. longipectoralis*	16,609	26.8	26.2	31.1	15.9	57.9	0.07	–0.24		OQ998928	
21	*T. longliensis*	16,570	27.3	25.5	31.4	15.8	58.7	0.07	–0.23		OQ998931	
22	*T. markehenensis*	16,569	28.7	25.3	28.2	17.8	56.9	–0.01	–0.17		KT213594	
23	*T. microps*	16,571	28.3	25.5	28.0	18.3	56.3	–0.01	–0.16		KT213595	
24	*T. moquensis*	16,571	28.6	25.3	28.4	17.7	57.0	0.00	–0.18		KT213597	
25	*T. nandanensis*	16,604	27.0	25.9	31.2	15.9	58.2	0.07	–0.24		OQ998932	
26	*T. nanpanjiangensis*	16,558	28.2	24.7	31.3	15.8	59.5	0.05	–0.22		OQ274895	
27	*T. nasobarbatula*	16,605	26.8	26.1	31.1	15.9	57.9	0.07	–0.24		MT361978	
28	*T. nujiangensa*	16,570	28.2	25.5	28.1	18.1	56.3	0.00	–0.17		KT213598	
29	*T. orientalis*	16,570	28.3	25.8	27.7	18.1	56.0	–0.01	–0.18		KT213599	
30	*T. pappenheimi*	16,572	28.7	25.0	28.7	17.5	57.4	0.00	–0.18		KT213600	
31	*T. pseudostenrua*	16,638	28.6	25.0	28.8	17.6	57.4	0.00	–0.17		KT213601	
32	*T. robusta*	16,570	28.4	25.3	28.2	18.0	56.6	0.00	–0.17		KM406486	
33	*T. rosa*	16,585	27.3	25.3	31.8	15.6	59.1	0.08	–0.24		JF268621	
34	*T. scleroptera*	16,570	28.5	25.4	28.2	17.8	56.7	–0.01	–0.18		KT213602	
35	*T. sellaefer*	16,574	28.8	25.2	28.1	18.0	56.9	–0.01	–0.17		KT213603	
36	*T. siluroides*	16,571	28.7	25.0	28.8	17.5	57.5	0.00	–0.18		JQ663847	
37	*T. stenura*	16,571	28.4	25.4	27.9	18.3	56.3	–0.01	–0.16		KY851112	
38	*T. stewarti*	16,567	28.3	25.5	27.8	18.5	56.1	–0.01	–0.16		KT213605	
39	*T. stoliczkai*	16,568	28.8	25.2	28.1	17.9	56.9	–0.01	–0.17		KT213604	
40	*T. strauchii*	16,590	28.5	25.4	28.3	17.8	56.8	0.00	–0.18		KP297875	
41	*T. tenuis*	16,571	28.2	25.7	27.5	18.6	55.7	–0.01	–0.16		KT224363	
42	*T. tianeensis*	16,573	27.1	25.9	30.5	16.4	57.6	0.06	–0.22		OQ998933	
43	*T. tibetana*	16,573	28.3	25.7	26.9	19.1	55.2	–0.03	–0.15		KT224364	
44	*T. ulacholica*	16,568	28.5	25.4	28.1	18.0	56.6	–0.01	–0.17		KT259194	
45	*T. venusta*	16,574	26.9	26.9	27.8	18.4	54.7	0.02	–0.19		KT008666	
46	*T. wuweiensis*	16,681	28.2	25.7	28.0	18.1	56.2	0.00	–0.17		KT224365	
47	*T. xiangxiensis*	16,598	26.8	26.3	30.8	16.0	57.6	0.07	–0.24		KT751089	
48	T. yangi*	17,068	27.5	25.7	30.4	16.4	57.9	0.05	–0.22		PQ356185*	
49	*T. zhenfengensis*	16,567	27.6	25.5	30.5	16.4	58.1	0.05	–0.22		OQ998934	
50	*Homatula potanini*#	16,571	26.3	26.9	30.2	16.6	56.5	0.07	–0.24		KP749475^#^	

*, the sequence obtained in this study; #, the sequence used as the outgroup

The dataset for further analysis comprised 49 mitogenome sequences, including 48 downloaded from NCBI and one obtained from this study. Total sequence lengths ranged from 16,558 to 17,068 bp, with A + T content exceeding 50% across all sequences. Among these, ten species exhibited positive AT-skew patterns, while the remainder showed negative values. All 49 species displayed negative GC-skew patterns (Table 2). After removing stop codons from each PCG, the final aligned length of the 13 concatenated PCGs was 11,400 bp. The Ka/Ks ratios for all 13 PCGs were less than 1, with the highest ratio (0.108) in ATP8 and the lowest (0.024) in COII. ATP8, ND2, and ND4 were found to have relatively fast evolutionary rates, while COI, COII, and COIII displayed slower rates (Fig. 3C). A 25-bp sliding window analysis of these PCGs revealed nucleotide diversity (Pi) variabilities across gene regions and species groups, with the highest Pi values in the surface-dwelling fish group (I), followed by the semi-cave-dwelling group (II), and then the cave-restricted group (III) (Fig. 3D).

### Sequence similarity analysis of *Triplophysa* species

**Fig. 4:**
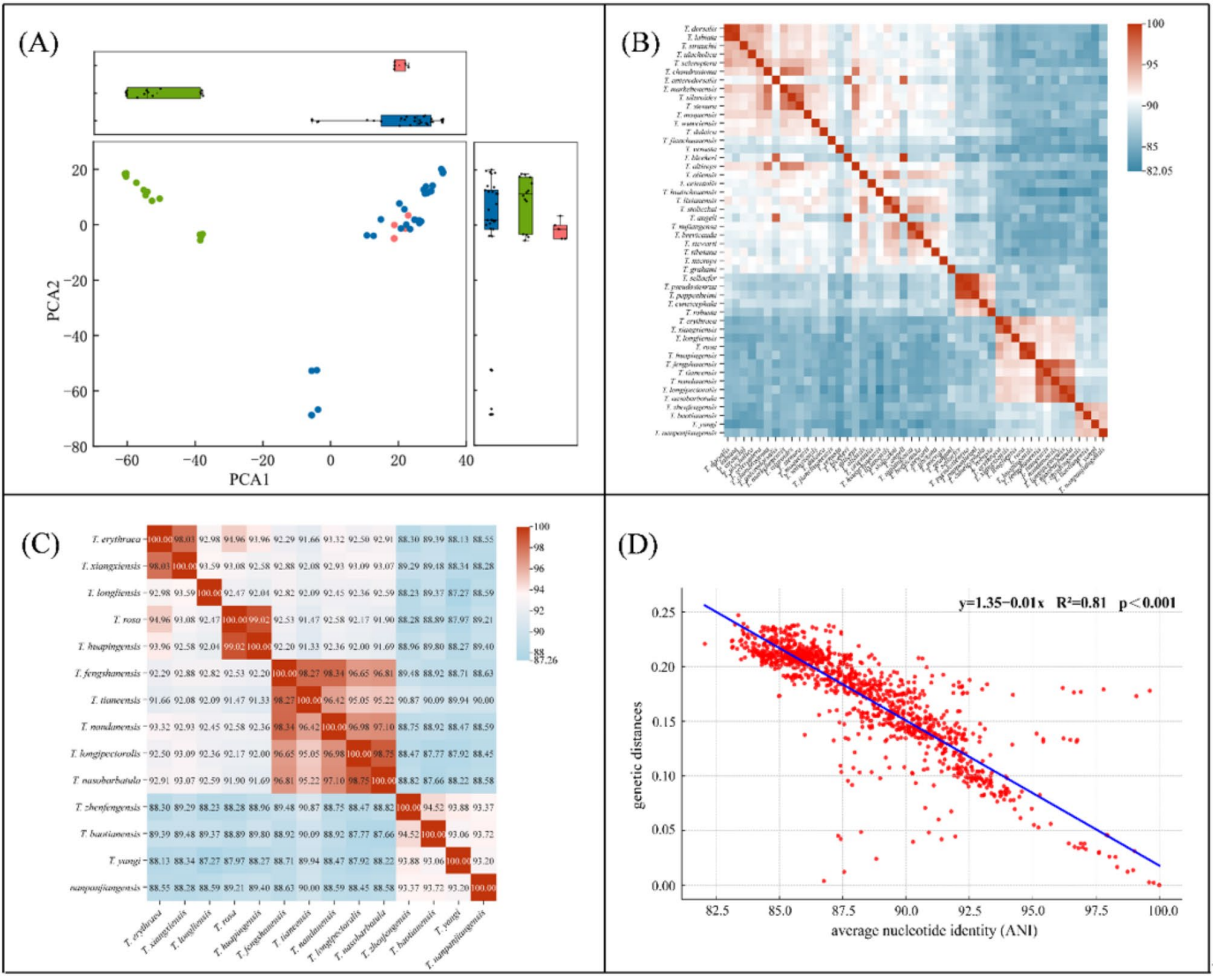
The sequence clusters and similarities analysis based on PCGs of *Triplophysa.* Notes: (**A**) The PCA plot determined by K-means clustering of 49 mitogenomes in *Triplophysa* (green, cave-dwelling groups; pink and red, surface-dwelling groups); (**B**) ANI plot of 49 mitogenomes in *Triplophysa*; (**C**) ANI plot and values within the 14 species in cave-dwelling groups (II & III). (**D**) Correlation plot between the phylogenetic distance and ANI values

The Kimura-2-parameter (K2P) distances of the COI sequences among 49 *Triplophysa* species ranged from 7.32 to 18.63%, while those of the concatenated PCGs ranged from 8.22 to 24.72%. *T. yangi* exhibited the smallest genetic distance with *T. baotianensis* based on both COI and concatenated PCGs analyses (Table S1). The PCA plot provided a reduced-dimensional view of the sequence data, capturing major variations among the 49 mitogenomes of *Triplophysa*. It demonstrated that hypogean lineage species cluster together, separating from the epigean lineage; the epigean lineage appeared to be further divided into two subgroups according to the sequence variations (Fig. 4 A). ANI analysis also significantly distinguished hypogean from epigean lineages, while the two groups we defined (II and III) in the hypogean lineage mixed together (Fig. 4B). Within the hypogean lineage, *T. yangi* exhibited the highest ANI value with *T. zhenfengensis* (93.88%) and the lowest value with *T. longliensis* (87.27%) (Fig. 4C). Additionally, correlation analyses indicated a generally negative relationship between ANI and genetic distance (R² = 0.81, *P* < 0.001) (Fig. 4D).

### Phylogenetic analysis of *Triplophysa*

**Fig. 5:**
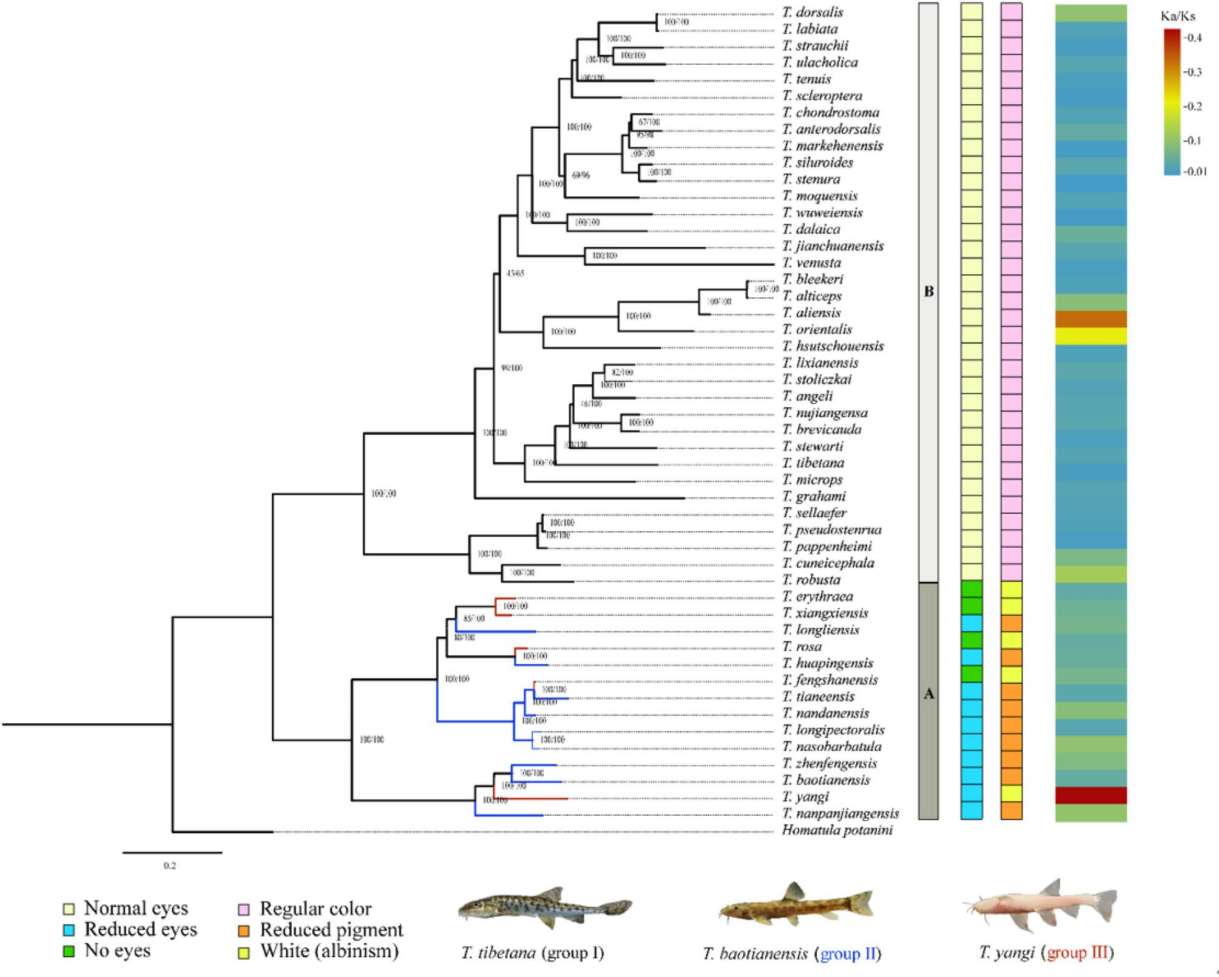
The phylogenetic relationship within *Triplophysa* based on 49 species. Notes: The numbers around the nodes are the bootstrap values and posterior probabilities from BI and ML methods. The species information used is listed in Table 2. Branches marked in black, green, and red indicate the species assigned into the surface-dwelling fish group (I), semi-cave-dwelling fish group (II), and cave-restricted fish group (III), respectively. The characteristics of eyes and body color, as well as the branch-wise Ka/Ks ratios are color-marked on the right, and the pictures of one representative species in each defined group are also provided

The phylogenetic trees derived from both BI and ML analyses exhibited identical topological structures, differing only in supporting values. Two major clades were identified with high support, confirming the established phylogenetic classification of epigean and hypogean lineages in *Triplophysa* [10], corresponding to Clade A (epigean) and Clade B (hypogean) in this study (Fig. 5). All species classified as group I based on morphological traits clustered within Clade A, while species in groups II and III formed Clade B. Notably, species in groups II and III intermixed without exhibiting mutually monophyletic structures. *T. yangi* displayed a sister group relationship with *T. zhenfengensis* and *T. baotianensis*, subsequently clustering with *T. nanpanjiangensis* to form an independent subclade. Interestingly, cave-restricted species from group III branched into several different clades within Clade B, indicating pervasive parallel evolution within the hypogean lineage.

### Selective pressures analysis on the mitogenomes of *Triplophysa*

The PAML branch test revealed an ω ratio of 0.0584 for the PCGs across all examined *Triplophysa* species, indicating overall constrained selection pressure. However, likelihood ratio tests (LRTs) indicated that both the two-ratio (M_2_) and three-ratio models (M_3_) provided a better fit than the one-ratio model (M_0_) (*P* = 0.000), suggesting variable ω ratios among certain groups. The three-ratio model was superior to one of the two-ratio models (M_3_ vs. M_2 − 1_, *P* = 0.000), but not significantly different from the other (M_3_ vs. M_2 − 2_, *P* = 0.062) (Table 3). These findings indicated that the group assignments in both M_3_ and M_2 − 2_ were similarly effective compared to M_2 − 1_. The ω ratios estimated under the free-ratio model (M_1_) further supported diverse selection pressures across branches, revealing significantly higher values for cave-dwelling species (II & III) compared to surface-dwelling species (I) (mean ω: 0.105 (II & III) vs. 0.064 (I); *P* = 0.001). Interestingly, *T. yangi* we sequenced here, as one typical species in the cave-restricted group, has the highest ω value of 0.4 among all the *Triplophysa* species examined in this study (Fig. 5). These results collectively suggested distinct ω ratios between surface-dwelling and cave-dwelling groups, with higher ω values in the latter. However, RELAX analysis indicated that cave-dwelling groups (II & III) experienced significant relaxation of selection compared to the surface-dwelling group (I), with K = 0.48 (*P* = 0.000).

According to the PAML site models, M_8_ performed better than M_7_ based on LRTs (M_8_ vs. M_7_, *P* = 0.000). Among 3,800 codon sites analyzed, 19 were identified under positive selection in the site model, while 67 sites were detected in the branch-site model. Seven sites from the site model and 13 from the branch-site model exhibited high Bayesian Empirical Bayes (BEB) values (> 0.95). Additionally, 18 and 27 sites were identified as under positive selection by FEL and MEME analysis, respectively. Five codon sites were identified by at least two methods, yielding a total of 31 codon sites considered potential positive selection sites with high credibility (Table 4). These sites were distributed across eight genes: ND1 (5), ND2 (6), ATP6 (2), ND3 (1), ND4 (4), ND5 (10), ND6 (1), and CYTB (2).

**Table 3:** Selection pressures estimation on PCGs of *Triplophysa* under branch model by codeml in PAML

Models	Code		lnL	Parameter estimates	Models compared	2∆L	*p*-value
One-ratio	M_0_	–122918.7780		ω = 0.04198			
Free-ratio	M_1_	–122711.2116		——			
Two-ratio (null)	M_2 − 1_	–122892.9577		ω0 = 0.04071, ω1 = 0.08528	M_2–1_ vs. M_0_	51.641	**0**
Two-ratio (null)	M_2 − 2_	–122858.5190		ω0 = 0.03825, ω1 = 0.07264	M_2–2_ vs. M_0_	120.518	**0**
Three-ratio (null)	M_3_	–122856.7836		ω0 = 0.03825, ω1 = 0.06838, ω2 = 0.08447	M_3_ vs. M_0_	123.989	**0**
Three-ratio (null)	M_3_	–122856.7836		ω0 = 0.03825, ω1 = 0.06838, ω2 = 0.08447	M_3_ vs. M_2–1_	72.348	**0**
Three-ratio (null)	M_3_	–122856.7836		ω0 = 0.03825, ω1 = 0.06838, ω2 = 0.08447	M_3_ vs. M_2–2_	3.471	**0.062**
Two-ratio (positive)	M_2 − 2 (p)_	–124664.3930		ω0 = 0.03681, ω1 = 1	M_2–2 (p)_ vs. M_2–2_	–3,611.748	**0**
Three-ratio (positive)	M_3 (p)_	–123289.4480		ω0 = 0.03791, ω1 = 0.06526, ω2 = 1	M_3 (p)_ vs. M_3_	–865.329	**0**

**Table 4:** Positive selection sites of high credibility on the PCGs of *Triplophysa* identified by codeml in PAML and HyPhy, and the amino acid properties changes identified by TreeSAAP

No.	*AA* positions	Gene names	Codeml in PAML			HyPhy			TreeSAAP		
			Site model, *PP* value (*P* < 0.05)	Branch-site model, *PP* value (*P* < 0.05)		FEL, P (*P* < 0.05)	MEME, P (*P* < 0.05)		Radical changes*	Total number	
1	10	ND1	—	0.999**		0.0127	—		*pK’*	1	
2	111	ND1	—	1.000**		—	—		*pK’*	1	
3	159	ND1	—	0.974*		—	—		*pK’*	1	
4	161	ND1	—	0.966*		—	—		*pK’*	1	
5	173	ND1	0.567	—		—	—		*pK’; R*_*a*_;	2	
6	85	ND2	0.998**	—		—	—		*pK’; α*_*m*_	2	
7	152	ND2	0.994**	—		—	—		*pK’; α*_*c*_; *R*_*α*_	3	
8	206	ND2	0.94	—		—	—		*P*_*α*_; *pK’;*	2	
9	228	ND2	0.903	—		—	—		*P*_*α*_; *pK’;*	2	
10	241	ND2	—	0.982*		—	—		*P*_*α*_; *P*_*β*_; *B*_*r;*_*pK’; R*_*a*_; *H*_*t*_	6	
11	318	ND2	1.000**			—	—		*P*_*α*_; *pK’; pH*_*i*_; *α*_*m*_; *R*_*α*_	5	
12	29	ATP6	—	0.949		—	—		*pK’*	1	
13	195	ATP6	—	0.999**		—	—		*pK’*	1	
14	87	ND3	0.933	—		—	—		*P*_*α;*_*pK’; α*_*c*_;	3	
15	91	ND4	0.672	—		—	—		*P*_*α*_; *c; pK’; α*_*m*_	4	
16	189	ND4	0.644	—		—	—		*pK’; α*_*c*_;	2	
17	194	ND4	0.574	—		—	—		*pK’; α*_*c*_;	2	
18	388	ND4	0.672	—		—	—		*pK’*	1	
19	2	ND5	—	0.863		—	0.01		*c; pK’; h; R*_*α*_	4	
20	33	ND5	0.980*	—		—	—		*P*_*α*_; *pH*_*i*_; *α*_*c*_; *α*_*m*_	4	
21	35	ND5	0.936	—		—	—		*P*_*α*_; *pK’; pH*_*i*_; *α*_*c*_; *α*_*m*_	5	
22	39	ND5	—	0.991**		—	—		*P*_*α*_; *pK’; pH*_*i*_; *α*_*c*_; *α*_*m*_	5	
23	120	ND5	0.844	—		—	—		*P*_*α*_; *pK’; α*_*c*_;	3	
24	275	ND5	0.718	—		—	—		*pK’; α*_*c*_	2	
25	277	ND5	0.861	—		—	—		*pK’; α*_*c*_	2	
26	506	ND5	—	0.519		—	0.04		*P*_*α*_; *B*_*r*_; *pK’; α*_*c*_; *R*_*α*_	5	
27	521	ND5	0.923	—		—	—		*P*_*α*_; *pK’; Mv; α*_*c*_; *R*_*α*_; *H*_*p*_	6	
28	523	ND5	0.794	—		—	—		*P*_*α*_; *pK’; R*_*α*_; *H*_*p*_	4	
29	119	ND6	0.777	—		—	—		*pK’*	1	
30	306	CYTB	—	0.986*		—	0.02		*P*_*β*_; *pK’; F; R*_*α*_; *H*_*t*_	5	
31	360	CYTB	—	0.999**		0.009	0.00		*pK’; H*_*p*_	2	

AA, amino acid; PP, posterior probabilities from Bayes empirical Bayes; *, Physicochemical amino acid properties available in TreeSAAP: α_c_: Power to be at the C-terminal; α_m_: Power to be at the middle of alpha-helix; B_r_: Buriedness; c: Composition; F: Mean r.m.s. fluctuation displacement; h: Hydropathy; H_p_: Surrounding hydrophobicity; H_t_: Thermodynamic transfer hydrophobicity; M_v_: Molecular volume; M_w_: Molecular weight; P_α_: α- helical tendencies; P_β_: β-structure tendencies; pH_i_: Isoelectric point; pK’: Equilibrium Constant of ionization for COOH; R_a_: Solvent accessible reduction ratio.

### Amino acid changes and structural analysis of positive selection sites

**Fig. 6:**
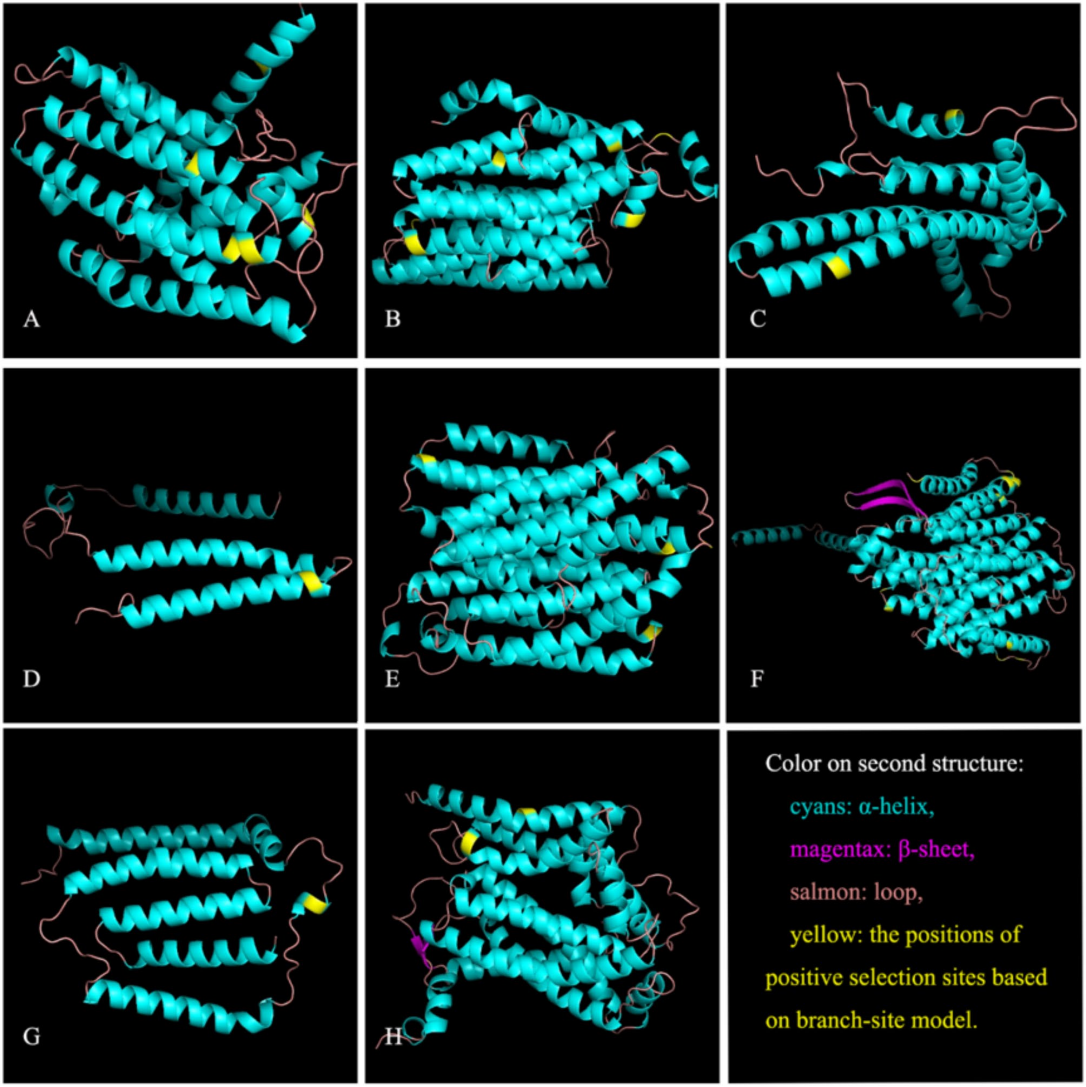
Positive selection sites highlighted in the crystal structure of ND1 (**A**), ND2 (**B**), ATP6 (**C**), ND3 (**D**), ND4 (**E**), ND5 (**F**), ND6 (**G**) and CYTB (**H**) based on the homologous protein. Notes: The sites with yellow color indicate the positions of potential positive selection sites of cave-dwelling species groups of *Triplophysa* under the branch-site model in PAML

All 31 potential positive selection sites of high credibility underwent radical substitutions in physicochemical properties, as determined by TreeSAAP (Table 4). Eleven sites exhibited four or more types of radical changes in amino acid properties. The positively selected sites identified in the branch-site models likely reflected the differences between cave-dwelling and surface-dwelling groups, as they were designated as foreground and background lineages. These sites were mapped onto the secondary and 3D structures of corresponding homologous proteins (Fig. 6). Most sites were located within functional domains of α-helices, particularly near or on the junction sites of α-helices and loop areas, which were probably crucial for the conformational stability of the relevant proteins.

## Discussion

### Mitogenome characteristics and gene duplications of *T. yangi*

Mitochondrial DNA is a powerful tool in molecular and evolutionary studies due to its advantages over complex nuclear DNA, playing crucial roles in reconstructing phylogenetic relationships, analyzing population genetics, and examining selective pressures [8, 20, 21]. In this study, we successfully sequenced and assembled the mitogenome of *T. yangi*, a newly identified cavefish exhibiting novel troglomorphic characteristics, with extraordinarily enlarged swim bladder chambers resembling a kind of “flotation device” (Fig. 1). The mitogenome length of 17,068 bp in *T. yangi* is the longest recorded among all *Triplophysa* species (Table 2). This length is attributed to an additional sequence inserted between ND2 and the WANCY region, comprising a large intact tandem repeat unit (A’-N’-OL’-C’) along with two unannotated flanking sequences (U1 & U2), totaling approximately 500 bp (Table 1; Fig. 2).

Typically, mitogenomes are compact with conserved gene order, and gene duplications in fish are rare [22]. Most duplications in fish species have been found around the control region, such as in *Muraenesox cinereus* [23], *Garra* cyprinids [24], Antarctic notothenioid fishes [25], and *Epinephelus* groupers [22]. In this study, we identified a new duplication pattern in the mitochondrial WANCY region, characterized by a core duplication unit with a large intact tandem repeat (A’-N’-OL’-C’) (Fig. 2). The fragments in this tandem repeat showed relatively low sequence similarities (86.15–93.55%) with the original copies (A, N, OL, C) of *T. yangi*, generally less than the genetic similarities between *T. yangi* and its sister species *T. baotianensis* at the COI gene (92.68%) and concatenated PCGs (91.78%). It suggested that the tandem repeat (A’-N’-OL’-C’) likely originated through one of two evolutionary pathways: (1) In situ duplication followed by long-term functional divergence (neofunctionalization or subfunctionalization) or pseudogenization, resulting in differential mutation rates between paralogous regions; (2) Horizontal acquisition from an exogenous source, possibly another *Triplophysa* species, with subsequent integration and functional co-option in the ancestral *T. yangi* mitogenome. However, current evidence cannot conclusively determine the predominant evolutionary mechanism driving this structural variation. Prioritizing integrated genomic and functional analyses will be essential to elucidate the underlying mitogenome plasticity in the future.

Gene rearrangements and duplications in mitogenomes may confer evolutionary advantages. Miya & Nishida [26] proposed a link between tRNA rearrangements and deep-sea adaptation, based on findings in *Gonostoma gracile*. Minhas et al. [25] discussed extensive duplications and rearrangements in Antarctic notothenioids, suggesting a role of mitochondrial gene duplication in cold adaptation, while He et al. [22] reported similar tRNA^Asp^ gene duplications in groupers, inferring lineage-specific adaptations. Unlike nuclear gene duplications, which are known to generate novel functions for adaptive evolution [27], the functional implications of mitochondrial gene duplications remain less understood [22]. Given mitochondria’s crucial role in the pathway of OXPHOS, any stable gene rearrangements may confer benefits for species adapting to complex and changing environments. As a typical cave-restricted species living in perpetual darkness, *T. yangi* likely faced distinct energy and metabolic demands. Thus, the unique intact tandem repeat pattern (A’-N’-OL’-C’) in its mitogenome may contribute somehow to its cave adaptation.

### Phylogenetic implications of *Triplophysa*

Phylogenetic relationships within the genus *Triplophysa* have garnered significant interest due to their species diversity and wide distribution across the Qinghai-Tibetan Plateau. However, reconstructing the complete phylogenetic picture within this largest genus of Cypriniforms has been challenging due to limited species coverage and insufficient molecular data. Feng et al. [28] conducted a phylogenetic analysis of *Triplophysa stoliczkae* using multi-locus genes, revealing significant discrepancies between analyses based solely on mitochondrial or nuclear genes. This suggests that potential gene flow events in the evolutionary history of *T. stoliczkae* may be better resolved by integrating multiple genetic markers. Wang et al. [29] performed a phylogenetic analysis within *Triplophysa* and reported the new mitogenome of *T. labiata*, identifying four subclades within the species dataset they used. They also uncovered a sister group relationship between *T. labiata* and *T. dorsalis*. Similarly, Wang et al. [30] sequenced the mitogenome of *T. bombifrons*, revealing the four main subclade relationships and the sister group relationship between *T. bombifrons* and *T. tenuis*. These studies underscored the importance of mitogenomic sequences in reconstructing phylogenetic relationships within the *Triplophysa* genus, providing a robust framework for future research.

The phylogenetic relationships reconstructed in our study (Fig. 5) align closely with the latest analysis based on mitogenomes by Zhang et al. [18]. Our study incorporated two additional species, *T. yangi* and *T. erythraea*, the latter of which was also a cave-restricted species whose mitogenomic characteristics have been reported in another study [31]. The two major clades revealed in our analysis confirmed the previous classification of epigean and hypogean lineages in *Triplophysa* [10]. Notably, in the hypogean lineage, cave-restricted species appeared in multiple distinct branches, indicating pervasive parallel evolution, similar to patterns observed in cavefishes of *Sinocyclocheilus* [8].

Despite our phylogenetic tree including a substantial number of *Triplophysa* species with available mitogenomic data, the overall phylogeny of the genus remains unresolved. The species coverage in this study is still limited: approximately 48 out of about 180 species (26.7%) in the genus and 14 out of about 40 species (35%) in the hypogean lineage. Given this limited coverage, we did not estimate divergence times, although such estimates could provide insights into the biogeographic history of this fascinating group. Additionally, divergence time estimates can vary significantly depending on the calibration strategy used, as seen in previous estimates of the divergence time of the most recent common ancestor (MRCA) of *Triplophysa* species [28, 32], where various calibration points were employed due to the lack of fossil records.

### Estimation of the selective pressures within *Triplophysa*

Compared to the surface-dwelling fish group (I), both the semi-cave-dwelling (II) and cave-restricted fish groups (III) exhibited significantly higher mean ω (Ka/Ks) ratios for the 13 mitochondrial PCGs (Table 3). It suggested that the cave-dwelling groups (II and III) have experienced reduced purifying selection efficacy, resulting in the accumulation of more nonsynonymous mutations. A high ω value can arise from either positive selection, which fixes beneficial nonsynonymous mutations, or relaxed functional constraints, which reduce the effectiveness of purifying selection and allow deleterious mutations to become fixed [33]. While it is challenging to distinguish between these scenarios based solely on ω values, the RELAX analysis indicated that relaxed functional constraints were likely the primary contributors to the elevated ω ratios in the cave-dwelling groups (II and III). However, positive selection may also contribute, particularly at the specific potential positive selection sites identified in this study (Table 4). The harsh physiological conditions in cave environments might impose strong selective pressures on energy metabolism genes, including mitochondrial OXPHOS genes. Furthermore, cavefish were often susceptible to population bottlenecks due to restricted migration, resulting in a small effective population size (Ne). This demographic constraint can significantly increase the ω ratio as well. Thus, the higher ω values in cave-dwelling groups (II and III) may reflect a complex interplay between ecological and physiological selective pressures and demographic restrictions that lead to genetic drift, which in turn may relax selection.

Positive selection was often observed over short evolutionary periods and typically affected only a few sites, making it challenging to be detected amidst the ongoing purifying selection at most sites within a gene [34, 35]. To address this, we utilized PAML’s site and branch-site models, alongside HyPhy tools FEL and MEME, to identify codon sites potentially under positive selection. By emphasizing convergent results from these approaches, we identified high-credibility potential positive selection sites (Table 4). Our findings revealed evidence of signatures of positive selection at specific amino acid positions in eight mitochondrial OXPHOS genes: ND1 (5), ND2 (6), ATP6 (2), ND3 (1), ND4 (4), ND5 (10), ND6 (1), and CYTB (2). All identified sites underwent radical changes in amino acid properties, suggesting that the extensive parallel evolution of cave-dwelling fishes in *Triplophysa* involved significant genetic adaptations to extreme subterranean environments across the extensive Karst areas of South China.

## Conclusions

This study has presented the first assembly of the complete mitogenome of *Triplophysa yangi*, which is 17,068 bp in length, making it the longest recorded for the genus *Triplophysa*. Compared to the mitogenomes of other *Triplophysa*, about 500 bp additional sequence was identified from *T. yangi*, comprising a large intact tandem repeat unit (A’-N’-OL’-C’) and two unannotated flanking sequences (U1 and U2). Its evolutionary origin may involve either in situ duplication events with subsequent functional divergence, or horizontal acquisition from exogenous genetic materials. The cave-restricted species in the hypogean lineages of *Triplophysa* exhibited signs of parallel evolution within the hypogean lineage. Selective pressure analysis indicated that the hypogean lineage (cave-dwelling groups, II & III) have significantly higher nonsynonymous/synonymous substitution ratios (ω) compared to the epigean lineage (surface-dwelling group, I). The duplication of tRNAs of *T. yangi* and the potential positive selection sites identified in *Triplophysa* cavefish further indicate adaptive evolution in mitochondrial PCGs in response to extreme subterranean conditions.

## Materials and methods

### Sampling and sequencing of *T. yangi*

The specimen of *T. yangi* used in this study (voucher number JWS20221148) was collected in December 2022 from a subterranean tributary of the Nanpanjiang River drainage in Wulong Township, Shizong County, Yunnan Province, China. The fish was euthanized before handling, using a solution of MS-222 (Macklin, Shanghai, China) at a concentration of 40 to 50 mg/L. Muscle tissue was then collected and DNA was extracted using the DNeasy Blood and Tissue Kit (Qiagen, Hilden, Germany). A DNA library was prepared using the Agencourt AMPure XP-Medium Kit (Beckman Coulter, USA) and the AxyPrep Mag PCR Cleanup Kit (Axygen, Corning, USA). High-throughput paired-end sequencing was performed on the MGISEQ2000 platform (Complete Genomics and MGI Tech, Shenzhen, China), generating approximately 44 Gb of raw reads with a read length of 150 bp.

### Mitogenome assembly and structural analysis of *T. yangi*

The complete mitogenome of *T. yangi* was firstly assembled using MitoZ [36], and further confirmed by NOVOPlasty [37] and MitoFinder [38]. Initial annotation of the assembled mitogenome was conducted using the online server MITOS2 (available at http://mitos2.bioinf.uni-leipzig.de/index.py [39]). Further annotation and gene map plotting were performed with the web application MitoFish (available at http://mitofish.aori.u-tokyo.ac.jp [40]). The secondary structure of tRNAs was predicted using tRNAscanSE 1.21 [41]. Nucleotide composition and codon usage of PCGs were computed using MEGA 11.0 [42]. Strand asymmetry was assessed using the formulas AT-skew = [A– T] / [A + T] and GC-skew = [G– C] / [G + C] [43].

### Sequence characteristics and similarity analysis of *Triplophysa* species

All available mitogenomes of *Triplophysa* were downloaded from GenBank, and one representative sequence was selected for each species for subsequent analysis. The final dataset comprised 49 *Triplophysa* species, including the one sequenced in this study. Based on the classification of cavefish in *Sinocyclocheilus* [8], *Triplophysa* species were categorized into three major groups according to morphological characteristics: (I) surface-dwelling fishes with normal eyes and typical coloration; (II) semi-cave-dwelling fishes with reduced eyes and partial loss of pigmentation; (III) cave-restricted fishes, lacking eyes or possessing only tiny eye dots, exhibiting white coloration (albinism). Relative synonymous codon usage (RSCU), the ratio of nonsynonymous substitution rates (Ka) to synonymous substitution rates (Ks), and nucleotide diversity (Pi) were calculated using DnaSP 6 [44]. Genetic distances were estimated with MEGA using the Kimura-2 parameter (K2P) [45]. Subsequently, PCA was conducted on the 49 mitogenomes using a specialized Python script for mitochondrial DNA sequences [46]. Average nucleotide identity (ANI) values were determined based on phylogenetic tree classification through pairwise comparisons using fastANI with the parameter “--minFraction 0.8” [47]. Correlation analyses of phylogenetic distance and ANI values from the complete mitogenomes were conducted using the MRM package in R (v4.3.1) [48].

### Phylogenetic analysis

Using *Homatula potanini* as an outgroup, phylogenetic analysis was conducted on the mitogenomes of 49 *Triplophysa* species. The 13 PCGs were concatenated and aligned using the CLUSTALW algorithm in MEGA [42]. Phylogenetic analyses were performed using maximum likelihood (ML) and Bayesian inference (BI), with partitioning schemes and nucleotide substitution models selected based on the Akaike Information Criterion (AIC) using PartitionFinder 2 [49]. ML analysis was executed with RaxML 8.0.2 [50], conducting 10 runs with random additional sequences and generating bootstrap values based on 1,000 rapid bootstrap replicates. BI analysis was performed with MrBayes 3.2.6 [51], running for 2,000,000 generations and sampling every 1,000 generations. Posterior probabilities (PP) were calculated to produce a consensus tree after discarding the first 25% of samples as burn-in.

### Selective pressures analysis

In molecular evolution, the ratio of nonsynonymous substitution (Ka) to synonymous substitution (Ks) per site, denoted as ω (Ka/Ks), reflects the selective pressures acting on a gene during its evolution. When ω > 1, the gene shows signs of positive selection; ω = 1 indicates neutral selection; and ω < 1 suggests purifying selection. The codon-based maximum likelihood (codeml) method, implemented in the PAML 4 package [52], was used to estimate ω values. First, branch models assessed ω values among groups based on the three categories defined above, allowing different branches to have distinct ω. The one-ratio model (M_0_), proposing a single ω for all branches, served as the null hypothesis of no adaptive evolution. Next, the two-ratio model (M_2_), permitting different ω values for background and foreground branches, identified groups of interest (M_2 − 1_: group III vs. groups I & II; M_2 − 2_: groups II & III vs. group I). The three-ratio model (M_3_), allowing independent ω values for groups I, II, and III, explored selective pressures across species. Additionally, the free-ratio model (M_1_), estimating specific ω values for each branch, examined variation in selective pressures across all groups. Differences in ω values among the three groups were statistically tested using the Wilcoxon rank sum test in SPSS 24.0 (SPSS Inc., Chicago, IL). To quantify potential positive selection probability across sequence sites, we implemented paired site models (M_7_ and M_8_), allowing ω to vary among sites (M_7_: beta [0 < ω < 1]; M_8_: beta & ω [ω > 1]) to identify positive selection at each site. Finally, branch-site models were employed to examine selective pressures, allowing each lineage and site to have its own ω value. This approach facilitates detection of selective pressure magnitudes on foreground branches and identification of positively selected sites. The paired comparison models used were Model A vs. Model A null. Likelihood ratio tests (LRTs) evaluated significance between comparative models by calculating twice the log-likelihood difference (2∆L) based on the chi-square distribution, with degrees of freedom (df) representing the difference in free parameters between models.

In addition to positively selected sites identified by the site and branch-site models from PAML, we conducted further identification using the Fixed Effects Likelihood (FEL) and Mixed Effects Model of Evolution (MEME) methods available on the HyPhy online platform Datamonkey [53]. FEL uses the maximum likelihood approach to infer Ka and Ks at each site, assuming constant selection pressure throughout the phylogeny. Positive selection was inferred when the likelihood ratio test yielded *p* < 0.1. In contrast, MEME employs a mixed-effects ML method to examine whether individual sites were under positive selection without needing to specify branches a priori. Candidate sites for positive selection were identified when β+ > α, with a significant likelihood ratio test at *p* < 0.1.

Additionally, to assess changes in substitution ratios potentially resulting from relaxed functional constraints within specific branches, we utilized RELAX software [54] to evaluate natural selection strength among different groups. RELAX begins by fitting a codon model with three ω classes to the entire phylogeny (null model) and assesses for relaxed or intensified selection by introducing ‘k’ as a parameter of selection intensity, where k ≥ 0 and k = Log ω_test_/Log ω_reference_. A significant result with k > 1 or k < 1 indicates intensified or relaxed selection strength along the test branches, respectively.

### Amino acid changes and structural analyses

The TreeSAAP program [55] was used to examine whether the positively selected sites identified in PAML and HyPhy exhibited changes in amino acid physicochemical properties at the protein level. TreeSAAP assessed the impact of natural selection based on 31 structures and physicochemical properties by measuring goodness-of-fit values. A change in amino acid properties within the range of 6 to 8 indicated radical alterations, suggesting potential positive selection [56]. To further investigate whether the positively selected sites were located in critical functional domains of the protein, we downloaded homologous protein structures of *Triplophysa* mitogenomes from the UniProt website (http://www.uniprot.org/). We highlighted the positively selected sites detected by PAML, HyPhy, and TreeSAAP on their three-dimensional structures using PyMOL software (Schrödinger, New York, USA).

## Electronic supplementary material

Below is the link to the electronic supplementary material.

Supplementary Material 1

## Data Availability

The final complete mitogenome, along with annotated information, has been deposited in GenBank under accession number PQ356185 (https://www.ncbi.nlm.nih.gov/nuccore/PQ356185). All the analyses and findings of this study were based on this sequence and other sequences available in GenBank.
